# Paraprobiotics and Postbiotics of Probiotic *Lactobacilli*, Their Positive Effects on the Host and Action Mechanisms: A Review

**DOI:** 10.3389/fnut.2020.570344

**Published:** 2020-10-22

**Authors:** Tsegay Teame, Anran Wang, Mingxu Xie, Zhen Zhang, Yalin Yang, Qianwen Ding, Chenchen Gao, Rolf Erik Olsen, Chao Ran, Zhigang Zhou

**Affiliations:** ^1^China-Norway Joint Lab on Fish Gastrointestinal Microbiota, Feed Research Institute, Chinese Academy of Agricultural Sciences, Beijing, China; ^2^Tigray Agricultural Research Institute, Mekelle, Ethiopia; ^3^AgricultureIsLife/EnvironmentIsLife and Precision Livestock and Nutrition Unit, AgroBioChem/TERRA, Gembloux Agro-Bio Tech, University of Liege, Passage des Deportes, Gembloux, Belgium; ^4^Norway-China Fish Gastrointestinal Microbiota Joint Lab, Institute of Biology, Norwegian University of Science and Technology, Trondheim, Norway; ^5^Key Laboratory for Feed Biotechnology of the Ministry of Agriculture and Rural Affairs, Feed Research Institute, Chinese Academy of Agricultural Sciences, Beijing, China

**Keywords:** paraprobiotics, postbiotics, *Lactobacilli*, metabolites, immunomodulatory effect

## Abstract

*Lactobacilli* comprise an important group of probiotics for both human and animals. The emerging concern regarding safety problems associated with live microbial cells is enhancing the interest in using cell components and metabolites derived from probiotic strains. Here, we define cell structural components and metabolites of probiotic bacteria as paraprobiotics and postbiotics, respectively. Paraprobiotics and postbiotics produced from *Lactobacilli* consist of a wide range of molecules including peptidoglycans, surface proteins, cell wall polysaccharides, secreted proteins, bacteriocins, and organic acids, which mediate positive effect on the host, such as immunomodulatory, anti-tumor, antimicrobial, and barrier-preservation effects. In this review, we systematically summarize the paraprobiotics and postbiotics derived from *Lactobacilli* and their beneficial functions. We also discuss the mechanisms underlying their beneficial effects on the host, and their interaction with the host cells. This review may boost our understanding on the benefits and molecular mechanisms associated with paraprobiotics and probiotics from *Lactobacilli*, which may promote their applications in humans and animals.

## Introduction

The genus *Lactobacillus* is the largest genus among lactic acid bacteria (LAB), consisting of more than 237 species (1), with continuous new species discoveries, such as *Lactobacillus metriopterae* (2) and *Lactobacillus timonensis* (3). Some *Lactobacillus* species are among the most widely used probiotics (4). Accumulating evidences are proposing that probiotic cell components or metabolites which interacting with the host cells may trigger probiotic effects (5–9). The advantages of metabolites and cell components of these probiotic bacteria over probiotic bacteria were clarified (10, 11). Furthermore, it has been reported that not all probiotic bacteria are safe. Concerns associated with live probiotic bacteria administration have been described in case reports, clinical trials and experimental models (12–14). Therefore, the applications of cell components or metabolites derived from probiotic strains are gaining more interest.

Regarding the use of cell components and metabolites of probiotics, different terms have been proposed, such as “paraprobiotics,” “ghost probiotics” “inactivated probiotics” “non-viable microbial cells,” “metabolic probiotics” “postbiotics,” etc. The concept of paraprobiotics was proposed to indicate the use of inactivated microbial cells or cell fractions that confer health benefit to the host (15). In some studies, cell wall components of the probiotics are categorized as paraprobiotics (16). Postbiotics are defined as soluble products or metabolites secreted by probiotics that have physiological benefits to the host (9). Similar definition as “factors resulting from the metabolic activity of a probiotic or any released molecules capable of conferring beneficial effects to the host in a direct or indirect way” was made by other researchers (17). To better differentiate cellular structural components and metabolites of probiotic strains, we define the cell structural components (mainly cell wall components) as paraprobiotics and secretory metabolites/componnets as postbiotics in this review.

The potential health benefits of probiotic *Lactobacillus* species isolated from the intestine of humans and animals have been documented in a plethora of research publications to date. The terms of paraprobiotics and postbiotics have emerged recently, but they have been adopted rapidly in several study areas including food science, food microbiology, and health and nutrition of human and animals. However, knowledge on the types of paraprobiotics and postbiotics is limited and some aspects related to the bioactivities and the action mechanisms of health-promoting effects of paraprobiotics and postbiotics remain unclear. The present review aims to update the evidence on the paraprobiotics and postbiotics derived from *Lactobacilli*, their physiological benefits and mechanism of interaction with the host cells.

## Isolation and Purification of Paraprobiotics and Postbiotics

Scientific evidences showed that there are different methods to isolate and purify paraprobiotics and postbiotics from several *Lactobacilli* species. Isolation of paraprobiotics and postbiotics from different probiotic bacteria involve cell disruption techniques including thermal treatment (18, 19), enzymatic treatments (60), solvent extraction (20), radiation (ionizing and UV rays) (21), high pressure (22) and sonication (23–26). Several other methods also have the potential to be used for production of paraprobiotics and postbiotics, such as ohmic heating and supercritical CO_2_, drying, pulsed electric field (PEF), and pH changes (27).

During the production of paraprobiotics from probiotics, it is important to expose the cells to factors (27) without disrupting cell structure (9). On the other hand, to isolate intracellular postbiotics, it is required to disrupt the bacterial membrane via combined treatments in order to obtain the intracellular metabolites (9). Furthermore, extraction and clean-up steps have been applied to help the isolation procedures, such as centrifugation, dialysis, lyophilization and column purification (23, 28–30). Secreted postbiotics by viable cells can be recovered from supernatants, and the viable cells can be eliminated from the medium by centrifugation and/or filtration (31). In most of the time, we can isolate paraprobiotics and postbiotics. However, in some cases it is difficult to separate them, and additional steps such as microfiltration are necessary to isolate the postbiotic fraction. The choice of techniques for isolation of postbiotics and paraprobiotics depend on the characteristics of molecules under study (32). Since the health benefits of paraprobiotics and postbiotics are influenced by their isolation methods, it is important to select the best methods and conditions for probiotic inactivation to obtain paraprobiotics and postbiotics (33).

## Categories, Properties, and Positive Effects of Paraprobiotics and Postbiotics Derived From *Lactobacillus*

Studies described that most of the paraprobiotics are located in the bacterial cell-envelope (5, 34). Generally, paraprobiotics consist of a wide range of molecules including peptidoglycans, surface proteins, cell wall polysaccharides, while postbiotics include secreted proteins and peptides, bacteriocins, organic acids, etc (10, 35–37). Furthermore, the paraprobiotics and postbiotics mediate a wide range of positive effects on the host such as immunomodulatory, anti-tumor, barrier-preservation, and antimicrobial properties (24, 38). Different species of *Lactobacillus* have different types of paraprobiotics and postbiotics. In the following part, we summarized the chemical composition and beneficial functions of paraprobiotics and postbiotics derived from *Lactobacilli* (Table 1).

**Table 1:** Probiotic effects of paraprobiotics and postbiotics in *Lactobacilli*.

**Probiotic effects**	**Paraprobiotics/postbiotics**	**Model**	**References**
Immunomodulatory effects	Peptidoglycan	Mice	(39)
	Teichoic acid	Porcine intestinal epithelial cell line	(7)
	Cell-wall polysaccharides Exopolysaccharides S-layer proteins	Mice and human cell lines Human cell lines Mice, 3T3 cells, Mouse cells	(40–42) (8, 43) (39, 44, 45)
	Pili proteins	Human cell, Murine cell lines	(46–48)
	Oligodeoxynucleotide (ODN)	Human cell lines	(49)
	Pyroglutamic acid dipeptides	Mouse cell lines	(50)
	Serine-Threonine peptide	Human cell lines	(51)
	Bacteriocins	Human and Mouse cell lines	(52–54)
	Short chain fatty acids	Mouse cell lines	(55); (56)
	Trp-Indole derivatives	Mouse cell	(57, 58)
	Conjugated linoleic acids	Human epithelium cell lines	(59)
Antagonistic effects against pathogens	Cyclic dipeptides	Human cells	(60)
	Bacteriocins	Human, Mouse cells	(61); (62)
	Conjugated linoleic acids	Human epithelium cell lines	(59)
Anti-tumor effects	Exopolysaccharides	Human colon cancer HT-29 cell	(63, 64)
	Conjugated linoleic acid S-layer proteins	Human epithelium cell lines, human prostate cancer cell line Human HT-29 cell line	(65); (66) (67); (68)
Preservation of intestinal barrier	LPXTG proteins	Human HT-29 cell line	(69)
	S-layer proteins	Human HT-29 cell line	(70)
	Moonlighting proteins	Human intestinal cell lines	(71)
	Pili proteins	Caco-2 cell line	(46, 72)
	Aggregation-promoting factor	Caco-2 epithelial cell lines	(73)
	p40 and p75 proteins	Mouse cell lines	(74, 75)

### Paraprobiotics

**Figure 1:**
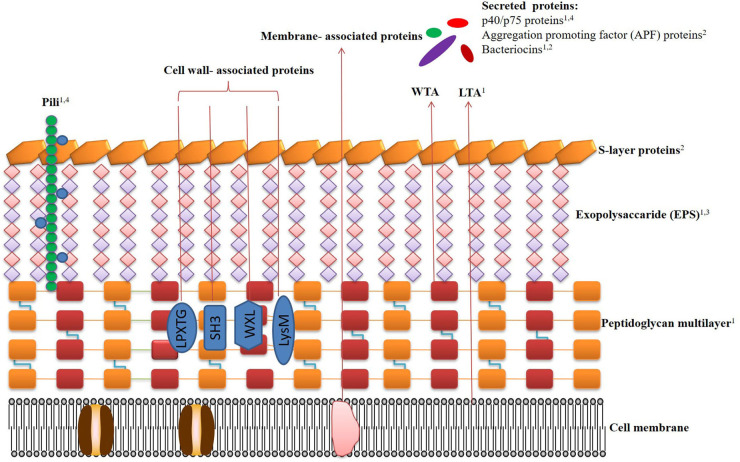
Schematic representation of the cell surface architecture of *Lactobacilli*, the bilipidic cell membrane (CM) with embedded proteins is covered by a multilayered peptidoglycan (PG) shell decorated with lipoteichoic acids (LTA), wall teichoic acids (WTA), pili, proteins, and lipoproteins. Exopolysaccharides (EPS) form a thick covering closely associated with PG and are surrounded by an outer envelope of S-layer proteins. The beneficial effects of the paraprobiotics and postbiotics are denoted by numbers. (1) immunomodulatory effects; (2) antagonistic effects against pathogens; (3) anti-tumor effects; (4) preservation of intestinal barrier. Related references are as follows. **Pili**: immunomodulatory effects (46–48), preservation of intestinal barrier (46, 72). **Protein p40/p75**: immunomodulatory effects (74), preservation of intestinal barrier (17, 75–78). **Aggregation promoting factor (APF) proteins**: antagonistic effects against pathogens (79–83). **Bacteriocins**: immunomodulatory effects (9, 84–87), antagonistic effects against pathogens (88–91). **LTA**: immunomodulatory effects (7, 92, 93). **Peptidoglycan**: immunomodulatory effects (39, 94, 95). **S-layers proteins**: antagonistic effects against pathogens (96–98). **Exopolysaccharides (EPS)**: immunomodulatory effects (99–102), anti-tumor effects (75, 103–106).

Studies confirmed that cell surface components of *Lactobacilli* are considered as an important part of effector molecules, as this part of the microbial cell is the first to interact with host cells. The cell envelope components of *Lactobacilli*, here categorized as paraprobiotics, include peptidoglycan, teichoic acid, cell-wall polysaccharides, cell surface-associated proteins, and proteinaceous filaments, which have been reported to mediate beneficial effects to the host (Figure 1).

#### Peptidoglycan

The cell wall of *Lactobacilli* contains a thick peptidoglycan layer, which is a multilayer, cross-linked glycan chain with a repeating pentapeptide unit of β-1,4-linked *N*-acetylglucosamine and *N*-acetylmuramic disaccharide units (107) and the fundamental composition of the glycan strands and pentapeptides was strain-specific for *Lactobacilli* (108). At the time of biosynthesis, assembly, and incorporation of peptidoglycan components, modifications happen in the bacterial peptidoglycan which could enhance the sensitivity to autolysis, hydrophobicity of the cell envelope, and resistance to lysozyme (109).

Peptidoglycan of *Lactobacillus casei* (*L. casei*), *Lactobacillus johnsonii* (*L. johnsonii*) JCM 2012 and *Lactobacillus plantarum* ATCC 14917 was reported to suppress interleukin-12 (IL-12) production via Toll-like receptor 2 (TLR2) which have been associated with autoimmune and inflammatory bowel diseases (94). Purified peptidoglycan from *Lactobacillus salivarius* (*L. salivarius*) Ls33 also exerted anti-inflammatory properties by inducing IL-10 production. Moreover, Ls33 peptidoglycan stimulated dendritic cell and T-cell regulatory functions upon sensing of nucleotide-binding oligomerization domain protein 2 (NOD2), and rescued mice from colitis induced by trinitrobenzene sulfonic acid (TNBS) (95). Furthermore, peptidoglycan from *Lactobacillus rhamnosus* (*L. rhamnosus*) CRL1505 was able to improve innate and systemic adaptive immune responses in mice (39). Notably, strain- or species-specific modifications of the conserved peptidoglycan polymers, including amidation, acetylation, and glycosylation, can lead to specific immunomodulatory capacities, which may contribute to the strain-specificity of probiotic effect.

#### Teichoic Acid

Teichoic acids (TAs) are the second main constituent of cell walls of *Lactobacilli* and account for up to half of the cell wall dry weight (110). Due to the anionic polymers nature of the TA, it can be covalently linked to peptidoglycan as wall teichoic acid (WTA) or anchored to the cytoplasmic membrane by their lipid anchors as lipoteichoic acid (LTA) (111).

Plethora studies reported the immunomodulatory characteristics of TA from many species of *Lactobacillus* (112). *L. plantarum* LTA (Lp.LTA) attenuated the expression of IL-8 induced by Pam2CSK and exerted anti-inflammatory effects on human intestinal epithelial cells (92). LTA of *L. plantarum* also showed anti-inflammatory responses in porcine intestinal epithelial cells (7). The anti-inflammatory functions and effects of LTAs are species or strain-specific. For instance, it has been shown that the majority of immunomodulatory properties induced by *L. plantarum* TA were dependent on D-alanylation (93).

#### Cell-Wall Polysaccharides

Polysaccharides are common in gram-positive bacteria surface including *Lactobacilli*. The most studied polysaccharides are exopolysaccharides (EPS). EPS may facilitate the interaction of the bacteria with the environment, mediate adhesion properties, protect against pathogens, and also act as a protective layer (43, 113).

Studies revealed that EPS derived from several species of *Lactobacillus* has a capacity to modulate systemic and mucosal immune responses, and provide direct health-promoting benefits. Purified EPS produced by *L. rhamnosus* RW-9595M exhibited immuno-suppressive effect on macrophages by inducing high levels of IL-10 and low or no tumor necrosis factor alpha (TNF-α), IL-6, and IL-12 (99). Moreover, the EPS-producing *L. plantarum* BGCG11 strain showed anti-inflammatory effect, pointing to an immune-suppressive role of EPS (100). Acidic fraction of EPS produced by *L. plantarum* 14 was able to decrease the production of pro-inflammatory cytokines (IL-6, IL-8, and MCP-1) in porcine intestinal epithelial cells in response to enterotoxigenic *Escherichia coli* (*E. coli*) (ETEC) challenge (101). Apart from the anti-inflammatory effect, EPS can also stimulate the immune response. EPS derived from yogurt fermented with *Lactobacillus delbrueckii* (*L. delbrueckii*) subssp. *bulgaricus* OLL1073R-1 induced interferon gamma (IFN-γ) production and activated natural killer (NK) cells in mice (102), which contributed to anti-viral infection effect (114). EPS can also regulate the energy metabolism of host. The EPS isolated from *L. rhamnosus GG* inhibited adipogenesis, and deceased the level of triacylglycerols and cholesterol ester in the liver and serum in mice (115).

Besides to the immunoregulatory effect of EPS, studies also described their anti-tumor abilities. *In vitro* anti-tumor assay of the EPS from *L. plantarum* YW32 proved their powerful inhibitory activity against colon cancer HT-29 cells (63). EPS isolated from *Lactobacillus acidophilus* 20079 strain can regulate both apoptotic and nuclear factor kappa B (NF-κB) inflammatory pathways in human colon cancer and have a potentiality to up-regulate the expression of IKbα, P53 and TGF genes (103). EPSs extracted from *L. casei* M5, *L. casei* SB27, *L. casei* X12, and *L. casei* K11 strains suppressed HT-29 cell growth via induction of G0/G1 cell cycle arrest and apoptosis (104). EPS from *L. plantarum* NCU116 induced c-Jun dependent Fas/Fasl-mediated apoptosis via TLR2 in mouse CT26 cells (105). Moreover, EPS from *L. acidophilus* inhibited the expressions of genes involved in tumor angiogenesis and survival of the colon cancer cell lines *in vitro* (106). Similarly, EPS from *L. acidophilus* LA1 demonstrated their anti-tumor activity *in vivo* against Ehrlich ascites carcinoma cells by suppressing the serum levels of malondialdehyde and nitric oxide (116) and EPS of *Lactobacillus gasseri* strains also showed their capability to inhibit cervical cancer cell growth and modulate immune response (117).

#### Cell Surface Proteins

Surface layer proteins are one of the most important components of the outermost cell envelope structures on *Lactobacilli* cell surface and other probiotic bacteria species. Cell surface proteins are classified as the proteins which are covalently or non-covalently attached to the cell surface. Recent study indicated that many types of surface proteins including LPXTG proteins, S-layer proteins, pili proteins, moonlight proteins are produced by *Lactobacillus* species including *L. plantarum, L. rhamnosus, Lactobacillus helvetics* (*L. helveticus*), and *L. acidophilus* (118). These proteins play significant positive roles on the host biological processes.

## LPXTG Proteins

LPXTG protein is one of the proteins covalently attached to the peptidoglycan of bacterial cell wall. These proteins contain a C-terminal LPXTG signal, and are linked to the cell wall by sortase A (SrtA). In *Lactobacilli*, LPXTG proteins are among the best-known covalent anchored surface proteins. LPXTG proteins were found in many *Lactobacillus* species including *L. plantarum* WCFS1, *L. johnsonii* NCC533, *Lactobacillus sakei* (*L. sakei*) 23K and *L. salivarius* UCC118 (119).

LPXTG proteins from different *Lactobacillus* species have been shown to bind to mucus and epithelial cells, and play major roles in bacteria-host interaction (120). About 12 proteins containing LPXTG motifs were identified from *L. plantarum*, which were involved in adhesion activity (120–122). Their major role was adherence to collagen, fibronectin, chitin, or mucus (123). Furthermore, studies with SrtA mutants of *L. casei* BL23 suggested that SrtA-dependent proteins participated in adhesion of this strain to Caco-2 and HT29 cells (69).

## S-Layer Proteins

Many *Lactobacilli* strains, including *Lactobacillus cripatus* (*L. crispatus*) ZJ001 and JCM 5810, *L. acidophilus* ATCC 4356, *Lactobacillus buchneri* (*L. buchneri*) CD034, and *Lactobacillus brevis* ATCC 8287, display a surface coating made of a crystalline, glycoprotein subunits also known as the S-layer (121). S-layer proteins are mostly anchored to peptidoglycan by non-covalent bonds (124). S-layer proteins of *Lactobacilli* account about 15% of total cell wall proteins and they differ from counterparts of other bacteria in their smaller size (25–71 kDa) and higher isoelectric point values (9.4–10.4) (125). In some species of *Lactobacillus*, S-layers with distinctive features can be found, such as glycosylated S-layers in *L. buchneri* and *Lactobacillus kefiri* (*L. kefiri*) (96).

Adhesive S-layers proteins of probiotic *Lactobacilli* can inhibit adherence and infection of pathogenic bacteria. S-layer proteins isolated from *Lactobacilli* were shown to bind to host cell proteins and extracellular matrix (44, 126). The S-layer protein from *L. kefiri* CIDCA 8348 improved the response of macrophages to lipopolysaccharide (LPS) (125) and was able to enhance the ovalbumin-specific immune response by triggering maturation of antigen presenting cells through the recognition of glycan moieties in mice (127). *Lactobacillus paracasei* subp. *paracasei, L. rhamnosus*, and *L. casei* strains isolated from natural dairy products are able to inhibit *Shigella sonnei* adhesion to HT-29 cells via their S-layer proteins (128). Similarly, the S-layer proteins from *L. helveticus* fb213, *L. acidophilus* fb116 and *L. acidophilus* fb214 contributed to the adhesion of the *Lactobacillus* strains to HT-29 cells and helped to inhibit the adherence and invasion of *E. coli* ATCC 43893 (96). S-layer proteins of *Lactobacillus* have also been demonstrated to competitively bind the intestinal epithelium *in vivo* and inhibit pathogen infection (97, 98).

## Pili Proteins

Pili are elongated protein structures protruding outside bacterial cells. Initially pili were considered as special features of pathogens (129), until they were found in *L. rhamnosus*. Pili bind to the intestinal mucusa and promote persistence of Lactobacillus strains in GI tract (130, 131). The SpaCBA pili of *L. rhamnosus* GG were a binding factor to human intestinal mucus, collagen, and intestinal epithelial cell (IEC) lines (46), and SpaC was credited as the major adhesion determinant (71, 107).

Studies also suggested other beneficial effects of pili derived from *Lactobacillus* strains. Mutant of *L. rhamnosus* GG devoid of SpaC induced increased mRNA expression of the pro-inflammatory cytokines IL-8 and TNF-α in Caco-2 cells while wild-type *L. rhamnosus* GG or SpaC alone had little impact on cytokine production (46). The immunomodulatory effect of SpaC was also observed in human fetal intestinal epithelial cell line H4 by modulating TLR-related gene expression (47). Comparative analysis of *L. rhamnosus GG* wild-type and isogenic pili mutants have shown immunoregulatory function of pilli by interactions with monocytes and dendritic cells (46, 48). Similar comparison also demonstrated that pilli can promote pathogen exclusion including pilliated *Enterococcus faecium* (132). Furthermore, SpaCBA pilli have been reported to be involved in promotion of cell proliferation in intestinal crypts, and protection against radiological insults (133). The SpaC pilin of *L. rhamnosus* GG (LGG) has been confirmed to induce the generation of reactive oxygen species (ROS) in epithelium and play a role in stimulating ERK phosphorylation and protecting the gut's epithelial barrier (133).

## Moonlighting Proteins

Moonlighting proteins include various classes of proteins, including translational elongation factors, metabolic enzymes, ribosomal proteins, and molecular chaperones (134–138). They are found in many species of *Lactobacillus* including *L. crispatus, L. plantarum, Lactobacillus reuteri* (*L. reuteri*) and *Lactobacillus jensenii* (*L. jensenii*) (135, 139–141).

Moonlighting proteins can mediate the colonization of the probiotic strains in intestinal tract. *L. acidophilus* used surface GAPDH to colonize the gut (142). *Lactobacillus* species including *L. plantarum, Lactobacillus fermentum* (*L. fermentum*), and *L. jensenii* were found to use moonlighting proteins in competitive exclusion and displacement of pathogens (140). Furthermore, moonlighting proteins including GAPDH, enolase and EF-Tu were involved in plasminogen/plasmin binding and activation (143), which might interfere with the exploitation of plasminogen by gastrointestinal pathogens that express plasminogen receptors or activators, such as *Helicobacter pylori* and *Salmonella* sp. (144).

### Postbiotics

As postbiotics, different secretory components of probiotic *Lactobacillus* strains have been reported to mediate beneficial effects, including proteins, peptides, organic acids, and other small molecules. These components can be secreted by live bacteria or released into the host environment after bacteria lysis and confer various physiological benefits to the host.

#### Secreted Proteins and Peptides

##### Protein p40 and p75

Protein p40 and p75 were identified from many *Lactobacilli* species including *L. casei, L. paracasei*, and *L. rhamnosus* (145). They are secreted cell wall muramidases and have approximately molecular sizes of 40 and 75 kDa, respectively (74). The positive contribution of these proteins secreted from *Lactobacillus* species has been described in several studies. The protein p40 from *L. rhamnosus* GG showed an immunomodulatry action in mice (74). The p40 transactivated the epidermal growth factor receptor (EGFR) in intestinal epithelial cells, inhibited apoptosis and preserved barrier function in the colon, thereby ameliorating intestinal injury and inflammation (17, 75, 76, 78, 109). Besides, p75 purified from *L. rhamnosus* GG and *L. casei* BL23 have anti-apoptotic activity by inducing the EGF/Akt pathway (145). Furthermore, the p40 and p75 proteins were able to protect the intestinal epithelial tight junctions and barrier functions by a protein kinase (PKC) and MAP kinase-dependent mechanism (76).

##### Aggregation-promoting factor (APF)

*Lactobacillus* species have been reported to secrete a number of aggregation promoting factor (APF) proteins, which are extracellular proteins responsible for bridging of conjugal pairs, self-aggregation, maintenance of cellular shape, and co-aggregation with other commensal or pathogenic bacteria (73, 82).

The function of APF from *Lactobacilli* mainly involves host colonization and pathogen exclusion. Previous studies demonstrated that *L. gasseri* SBT2055 decreased adhesion and invasion of *Campylobacter jejuni* (*C. jejuni*) *in vitro* and hindered its infection in chickens via co-aggregation with the pathogens, and the co-aggregation was mediated by proteinaceous cell-surface components (79). Similarly, Yungareva and Urshev (81) also confirmed that APF in *Lactobacillus delbr* (*L. delbr*). subspp *Bulgaricus* had co-aggregation property which inhibited the growth of pathogenic bacteria. APF-2 from *L. gasseri* ATCC 9857 strain contributed to inhibition of the adhesion of *Trichomonas vaginalis* to human vaginal ectocervical cells (80, 81). Furthermore, the presence of high concentration of intracellular GGDEF protein (DgcA) in *L. acidophilus* and a serine/threonine-rich APF protein from *L. plantarum* NCIMB 8826 resulted in increased production of EPS and enhanced the co-aggregation ability (82, 83). The aggregation phenotype enables *Lactobacilli* strains to colonize the GI tract, and to inhibit adhesion of pathogens by competitive exclusion or by co-aggregation with pathogens (62, 146).

##### Bacteriocins

Bacteriocins are a class of powerful small ribosomally synthesized antimicrobial peptides with bactericidal or bacteriostatic functions (147). Various types of bacteriocins were produced by *Lactobacilli* species, such as lactacin B from *L. acidophilus* and *L. johnsonii*, lactocin from *L. casei*, Lactocin 705 from *L. casei*, Lactoccin G from *L. lactis* and plantaricin from *L. plantarum* (148, 149).

Bacteriocins of probiotic *Lactobacilli* can mediate inhibitory effect against pathogens. Bacteriocin PJ4 produced by *L. helveticus* PJ4 isolated from rat gut microflora was active against enteric pathogen (88) and bacteriocin DT24 produced by vaginal *L. brevis* DT24 was antagonistic against uropathogenic *E. coli* (89). Pangsomboon et al. (150) reported that bacteriocins from *L. paracasei* were able to kill *P. gingivalis*. Moreover, the bacteriocin extracted from probiotic *L. acidophilus* KS40 was able to inhibit urogenital pathogens such as *Gardnerella vaginalis, Streptococcus agalactiae*, and *Pseudomonas aeruginosa* (90). Reuterin produced by *L. reuteri* (6) exerted antimicrobial effects by modifying thiol groups and inducing oxidative stress in bacterial cells (151). *L. salivarius* UCC118, a probiotic strain of human origin, produced bacteriocin Abp118, which mediated the inhibitory effect of the probiotic against *Listeria monocytogenes* infection in mice (91). Additionally, purified bacteriocins from different *Lactobacillus* species have shown anti-infective functions in mice models, demonstrating that bacteriocins can be a promising alternative against gastrointestinal infections (152).

Besides the antimicrobial effects, bacteriocins produced by *Lactobacillus* may also affect host immunity. Plantaricin was identified as the factor in *L. plantarum* WCFS1 that modulate the immune response of DCs (84). Notably, plantaricin can be produced during *L. plantarum* WCFS1 colonization in mice, thus supporting the function of this bacteriocin under *in vivo* conditions (85). Phagocytosis activities of macrophage were improved by bacteriocins isolated from *L. acidophilus* (87). Moreover, bacteriocins can affect the immune function of the host by selectively competing with specific bacterial strains and shaping the microbiota composition (9, 86).

#### Small Molecules

Small molecules differ from the above mentioned paraprobiotics and protein/peptide postbiotics in that they do not have strain-specific differences in the biochemical characteristics and therefore are generally not responsible for strain-specificity of probiotic functionality. Moreover, different from protein/protein postbiotics, they can be produced by strategies independent of the probiotic strains. However, subsets of the probiotic effects are mediated by small molecules. Therefore, in this review, we also categorized small molecules as postbiotics, and summarized their beneficial effects.

##### Short chain fatty acids

SCFAs are produced by gut microbiota from indigestible food components such as fiber, oligosaccharides and polysaccharides via different metabolism channels (153, 154). The SCFAs have a wide range of positive effects on the host, such as providing energy sources for colonic epithelium cells (155), maintaining metabolic homeostasis (156), regulating T regulatory cells (157, 158), and anti-inflammatory effects (159–162). Generally they are essential for the health and well-being of the host when present in sufficient amounts (163). Studies showed that *Lactobacillus* strains can produce different types of SCFAs. *L. rhamnosus* GG and *L. gasseri* PA 16/8 produce propionate (163, 164). Moreover, SCFAs have been associated with the beneficial effects of probiotic *Lactobacillus* strains in some research. Dhaliwal et al. (165) confirmed that supplementation of mice with *L. plantarum* showed an increase in acetate and butyrate levels and reduced intestinal permeability and monoamine oxidases in the brain. SCFAs-promoting probiotic *L. johnsonii* L531 treatment have been shown to control *Salmonella* infection and maintaining metabolic homeostasis in pig (166). In a screening of LAB to reduce cholesterol levels, the strain of *L. plantarum* CECT 7529, which produced higher quantities of propionic and butyric acids, showed excellent properties for reducing cholesterol levels (167). Furthermore, probiotic strains *L. salivarius* FP25 and FP35, and *L. reuteri* NCIMB exhibited inhibitory effect on colon cancer cell proliferation, which was mediated by the production of SCFAs (168, 169).

##### Conjugated linoleic acid (CLA)

Studies showed that many *Lactobacillus* species are able to synthesize conjugated linoleic acids (CLAs) (170, 171). The ability of *L. rhamnosus* PL60 to produce *cis*-9, *tra*-11 and *tra*-10, *cis*-12-CLA in humans was the first report indicating that probiotic bacteria produce CLA (172). Further studies showed that some Lactobacilli species isolated from GI tract of human and animals, including *L. rhamnosus, L. acidophilus* and *L. plantarum*, are CLA producers (173, 174).

CLA inhibited the growth of HT-29 and Caco-2 cancer cell lines *in vitro* (175). Proliferation of MDAMB-231 cells was inhibited by *L. plantarum*-produced CLA in a dose dependent manner (176). *In vivo* administration of CLA to rats could decrease the occurrence of colonic tumors and increase the apoptotic indices (177). Moreover, CLA has been shown to reduce the incidence of colonic, skin, mammary, and prostate carcinogenesis in animal models (178).

CLAs produced by probiotic *Lactobacillus* have remarkable anti-tumor effect. CLA inhibited the growth of HT-29 and Caco-2 cancer cell lines *in vitro* (175). Proliferation of MDAMB-231 cells was inhibited by *L. plantarum*-produced CLA in a dose dependent manner (176). *In vivo* administration of CLA to rats could decrease the occurrence of colonic tumors and increase the apoptotic indices (177). Moreover, CLA has been shown to reduce the incidence of colonic, skin, mammary, and prostate carcinogenesis in animal models (178).

##### Neurotransmitters

Gut bacteria contribute to the proper function of gut-brain axis by producing neurotransmitters, such as γ-aminobutyric acid (GABA), glutamate, serotonin (5-HT), dopamine (DA), norepinephrine, histamine and acetylcholine (179). Particularly, *Lactobacillus* can produce multiple neurotransmitters, such as GABA (180–186), serotonin (181), catecholamines (181), dopamine (181), and acetylcholine (187). Different probiotic *Lactobacillus* strains have been reported to confer beneficial effects on mental health, acting as “psychobiotics,” including *L. paracasei* (188), *L. helveticus* (189, 190), *L. plantarum* (165, 191), and *L. rhamnosus* (192). Moreover, studies have shown that histamine and dopamine produced by gut commensal *Lactobacillus* imparted significant role in sleep related disorders and regulates neuronal signaling in depression, anxiety related conditions disease (193), suggesting that the beneficial effects of probiotic *Lactobacillus* on mental health might be attributable to the neurotransmitters production.

## Interaction of Paraprobiotics and Postbiotics With Their Receptors on Host Cells

The beneficial effects of paraprobiotics or postbiotics are mediated through an interaction between the microbial products and host. Probiotic *Lactobacilli* possess conserved MAMPs, including peptidoglycan, LTA, S-layer protein A (SlpA), EPS, and genomic DNA, which can be recognized by pattern recognition receptors (PRRs), induce downstream signaling cascades that confer the beneficial functions (5).

**Figure 2:**
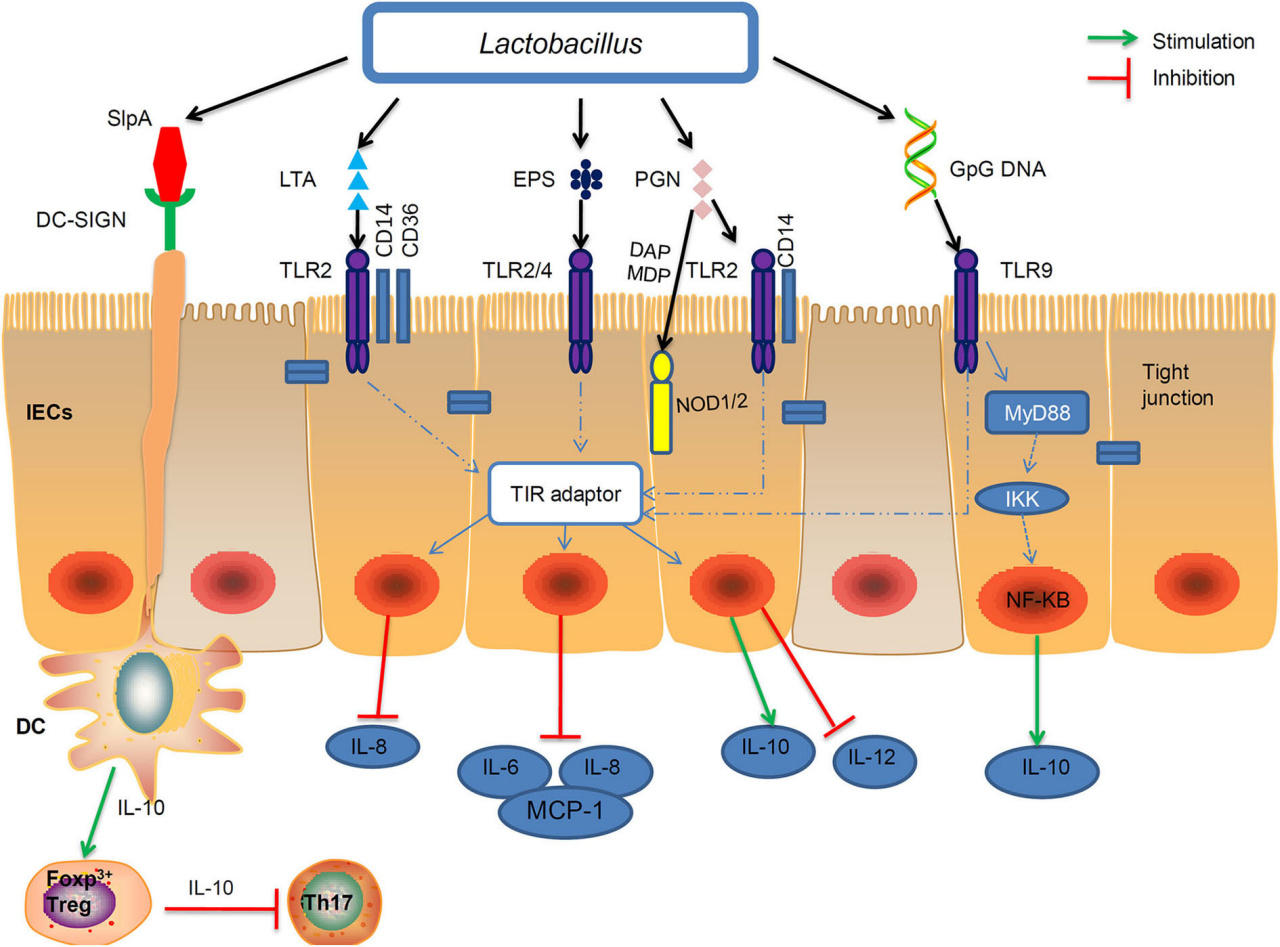
Interactions of the MAMP of *Lactobacillus* with PRRs of the epithelial and immune cells of the host. Probiotic *Lactobacillus* possess conserved microbe-associated molecular patterns (MAMPs), including peptidoglycan, lipoteichoic acids (LTA), S-layer protein A (SlpA), exopolysaccharides (EPS), and genomic DNA which can be recognized by certain pattern recognition receptors (PRRs). Peptidoglycan and LTA interact with TLR2. Moreover, specific components of peptidoglycan, such as meso-DAP and MDP, are recognized by NOD1 and NOD2, respectively. The EPSs of *L. delbrueckii* TUA4408L, act as TLR2 and TLR4 ligands to exert anti-inflammatory activities by inhibiting the production of IL-6, IL-8, and MCP-1. On the apical side of IECs, CpG-DNA stimulated TLR9 interacts with MYD88 and the inhibitor of NF-κB kinase (IKK) complexes, which may induced IL-10 expression. Binding of SlpA to the DC-SIGN (dendritic cell-specificICAM3-grabbing non integrin) receptor can induce IL-10 production in DCs and development of T cells. IEC, intestinal epithelial cell; DC, dendritic cell; Treg, T regulatory cell; Th, T helper cell; MCP-1, monocyte chemoattranctant protein-1.

The importance of Toll-like receptors (TLRs) and Nucleotide-binding oligomerization domain-like receptors (NLRs) in mediating differential host interaction with paraprobiotics and probiotics has been widely acknowledged (107, 194). In this review we summarize four types of the PRRs that play principal roles in the regulation of the host's immune response and these different types of PRRs can bind to specific paraprobiotics or postbiotics of *Lactobacillus* strains (Figure 2).

### Toll-Like Receptors (TLRs)

TLRs recognize distinct families of MAMPs. For instance, TLR2 recognizes LTA and peptidoglycan; TLR2/TLR4 recognize bacterial EPS with the help of RP105/DM1; TLR9 is responsive to unmethylated CpG oligonucleotide (CpG-ODN) (195) (Table 2). *L. reuteri* DSM 17938 strain showed a positive effect against necrotizing enterocolitis via TLR2 (203). TLR2 recognized the LTA of *L. plantarum*, and attenuated Pam2CSK4-induced IL-8 expression (46).

**Table 2:** Receptors, ligands, and immunological effects.

**Receptors**	**Ligands**	**Probiotic effects**	**Model**	**References**
**TOLL-LIKE RECEPTORS (TLRs)**				
TLR2	Peptidoglycan	Down-regulate IL-12	Mouse cell lines	(94)
TLR2	LTA	Down-regulate IL-8, balance IL-10/IL-12	Human epithelial Caco-2 cell line	(92, 196)
TLR2, TLR4, RP105/MD	EPS	Down regulate IL-6, IL-8, MCP-1,	Porcine intestinal cell lines	(101, 197)
TLR9	Unmethylated CpG DNA	Suppress NF-κB signaling pathway	Porcine cell lines, Mouse cell lines	(198, 199)
**NUCLEOTIDE-BINDING OLIGOMERIZATION DOMAIN-LIKE RECEPTORS (NLRs)**				
NOD1 and NOD2	Meso-DAP, MDP	Up-regulate IL-10, suppress the production of IL-12	Mouse cell lines	(94)
**C-TYPE-LECTIN RECEPTORS (CLRs)**				
DC-SIGN	SlpA	Up-regulate IL-10, IL-4	Human cell lines	(200)
**G-PROTEIN-COUPLED RECEPTORS (GPCRs)**				
GPR41 GPR43 GPR109A	Acetate, propionate, and butyrate	Down-regulate TNF-α, IL-6, IL-12, and NO up-regulate IL-10	Human cell lines	(201); (202)

The EPS of *L. delbrueckii* TUA4408L can act as TLR2 and TLR4 ligands, and exert anti-inflammatory activities in porcine IECs by modulating MAPK and NF-κB signaling pathways (197). *L. plantarum* N14 EPS reduced inflammation in intestinal epithelial cells depending on RP105/MD1 complex (a member of TLR family). (101). Similarly, *L. rhamnosus* GG and its components (surface layer protein and EPS) inhibited MAPK and NFκB signaling and alleviated LPS-induced inflammatory cytokines in porcine intestinal epithelial cells by modulating TLR expressions (204).

### Nucleotide-Binding Oligomerization Domain-Like Receptors (NLRs)

NLRs constitute a large family of PRRs and includes a number of subfamilies, which can be distinguished depending on the N-terminal effector domains (195). Two well-studied NLR proteins are NOD1 and NOD2. The NOD1 recognizes molecules containing D-Glu-mDAP (205), whereas NOD2 are vital for the regulation of NAM-D-Ala-D-Glu unit of the molecules (206). Recognition of muropeptide from *Lactobacilli* by NOD2 can induce anti-inflammatory properties and protect mice from colitis development (94). Different types of signaling molecules from *Lactobacilli* species including the fragments of peptidoglycan were sensed by NODs (207), and this sensing results in the activation of NF-κB and antimicrobial activity (208).

### C-Type Lectin-Like Receptors (CTLRs)

CTLRs recognize carbohydrates molecules, through one or more carbohydrate recognition domains (CRDs) (209). The sugar moieties found in the glycan backbone of the bacterial peptidoglycan bind CTLRs (210). After the ligand recognition, specialized CTLRs trigger or inhibit wide ranges of signaling pathways, thus modulate diverse immune responses (211).

DC-specific ICAM-3-grabbing nonintegrin (DC-SIGN) is a CLR expressed mainly on dendritic cells (DCs) and recognizes mannose- and fucose-containing glycans that are present on many species of *Lactobacilli* bacterial cell surfaces. DC-SIGN was previously shown to bind *L. acidophilus* SlpA *in vitro* (200). SlpA-DC-SIGN interaction induced IL-10 production in DCs promoted of T cells that secrete high amounts of IL-4, thereby decreasing the Th1/Th2 ratio (200). Further, *in vivo* role of the SlpA-induced protective immune regulation was demonstrated (212).

### G-Protein-Coupled Receptors (GPCRs)

The best characterized GPCRs are GPR41 and GPR43, which are highly expressed by epithelial cells, adipocytes, enteroendocrine cells and the cells of the sympathetic nervous system (213), and are mainly activated by SCFAs (214). Butyrate and propionate produced by microbiota in the gut acted with GPR43 and regulated the accumulation of Foxp3^+^ Treg cells (215). The recognition of SCFAs by GPR109A has also been reported. For instance, activation of the GPR109A receptor by butyrate induced the differentiation of regulatory and IL-10-producing T cells, which suppressed colonic inflammation and carcinogenesis by promoting anti-inflammatory properties in colonic macrophages and dendritic cells (216). Furthermore, SCFAs produced by gut microbiota may regulate lipid metabolism, glucose homeostasis and insulin sensitivity through GPCR signaling (156).

## Conclusions

Paraprobiotics and postbiotics derived from *Lactobacillus* species consist of a wide range of effector molecules. These products and byproducts of probiotic *Lactobacillus* have been found to possess magnificent beneficial functions including preservation of epithelial barrier, anti-tumor effect, immunomodulation, and antagonistic effects against pathogens. Furthermore, they have various advantages compared with probiotics, including clear chemical structures and safety dose parameters, as well as longer shelf life (217, 218). Therefore, the use of paraprobiotics and postbiotics may represent a valid and safer alternative to live probiotic bacteria, and have exhibited good potential to replace probiotics (219, 220).

The mechanisms underlying the beneficial effects have been less known, especially the signaling pathways downstream the interaction of paraprobiotics/postbiotics and PRRs, which deserve more investigation. Furthermore, the structure-activity relationship (SAR) of paraprobiotics and postbiotics will be an interesting topic, which may guide the functional improvement of these probiotic components, by either chemical or biological strategies.

Currently the application of postbiotics and paraprobiotics in human food, animal feed and pharmaceutical industries is increasing and several paraprobiotic and postbiotics products derived from *Lactobacill* species are commercially available for prevention or treatment of some diseases (221–225). Nevertheless, more evidence is needed to validate the beneficial effects of paraprobiotics and postbiotics. Current advancement of molecular technologies such as multi-omics have been promoting the identification of more paraprobiotics and postbiotics from probiotic *Lactobacillus* strains. Moreover, novel probiotics from other family or phylum are being discovered and studied, such as commensal bacterium isolated from the intestine of both human and animals (226, 227). The techniques and experience of paraprobiotics and postbiotics discovery from probiotic *Lactobacilli* may guide the investigation of novel functional components derived from the new probiotics. Collectively, paraprobiotics and postbiotics have good potential as prophylatctic or therapeutic agents as well as functional food or feed additives for human or animal use.

## Author Contributions

All authors listed have made a substantial, direct and intellectual contribution to the work, and approved it for publication.

## Conflict of Interest

The authors declare that the research was conducted in the absence of any commercial or financial relationships that could be construed as a potential conflict of interest.
